# The Impact of the Lubricant Dose on the Reduction of Wear Dies Used in the Forging Process of the Valve Forging

**DOI:** 10.3390/ma14010212

**Published:** 2021-01-04

**Authors:** Łukasz Dworzak, Marek Hawryluk, Marta Janik

**Affiliations:** 1 Department of Metal Forming, Welding and Metrology, Wroclaw University of Science and Technology, Lukasiewicza Street 5, 50-370 Wroclaw, Poland; marek.hawryluk@pwr.edu.pl; 2 Mahle Poland, Mahle 6, 63-700 Krotoszyn, Poland; marta.janik@pl.mahle.com

**Keywords:** lubrication, wear, cooling–lubrication system, valve forging, 3D scanning method

## Abstract

The paper presents the results of research on the influence of the settings of lubrication and cooling system parameters (solenoid valve opening time and lubricant feed pressure in terms of its quantity) in order to select the optimal lubricating conditions and thus reduce the wear of the dies used in the first forging operation of the valve forging made of high-nickel steel. Based on the observation of lubrication in the industrial process, it was found that a significant part of the lubricant fails to reach the die cavity, reaching the outside of it, which causes die wear due to seizure resulting from adhesion of the forging material to the tool surface as well as high lubricant consumption and dirt in the press chamber. The authors proposed their own mobile lubricating and cooling system, which allows for a wide range of adjustments and provided with automatic cleaning procedures of the entire system, unlike the fixed lubrication system used so far in the industrial process. First, tests were carried out in laboratory conditions to determine the highest wettability and the lubricant remaining inside the tool cavity. These tests determined the lubrication system parameter settings that ensured that the greatest amount of lubricant remains in the cold die cavity without the forging process. Then, to verify the obtained results, tests were carried out in the industrial process of hot die forging of valve forgings for short production runs of up to 500 forgings. The results were compared with the measurement of changes in the geometry of tools and forgings based on 3D scanning and surface topography analysis with the use of SEM (Scanning Electron Microscope). For the best results (the variant of the setting of the dose and the time of exposure to lubricant), the forging process was carried out with the use of a new tool up to the maximum service life.

## 1. Introduction

In the case of die forging processes, lubrication is of key importance to obtain forgings that are correct with respect to geometry and quality as well as the service life of forging tools. In hot die forging processes, dies and punches are subjected to very high and cyclic mechanical loads, even above 1500 MPa, and heat loads in the range of 60 to 800 °C, which results in high temperature gradients [1]. In particular, the heat load intensifies abrasive wear caused by high pressures, which translates into a reduced durability of tools and forging equipment [2,3,4]. Therefore, on the one hand, the lubrication and the use of an appropriate lubricant/coolant is required due to the necessity to minimize friction to accurately fill the working cavity of the tool by the deformed material [5,6]. On the other hand, the use of lubricant/coolant primarily reduces the friction between the deformed forging material and the die material, as well as isolates the working tool cavity from direct contact with the hot forging being deformed. Lubrication effectively reduces the tool surface temperature as well as the intensity of oxidation and tempering processes [7]. The disadvantage of lubrication is the cyclic cooling of the tool surface, which can intensify the thermal and thermomechanical fatigue process. Testing and analysis of the properties of lubricants used in forging processes at elevated temperatures is discussed extensively in the available literature [8,9,10,11,12]. Moreover, due to continuous technological development and competition, more and more tests and applications related to lubricants, dedicated to specific forging processes, apart from research in scientific centers, are offered by leading global lubricant manufacturers, including Acheson, Fuchs, Henkel/Bechem, Houghton, and Oelheld. Therefore, the application of the optimal lubricant involves considering all the often-opposing aspects and consequently choosing the right solution for the application. At the same time, it should also be emphasized that the correct/effective method of lubricating and cooling the forging tools is affected not only by lubricant properties and type but also the method and direction of its feeding as well as the amount and frequency of exposure of the lubricant dose. In the case of forging processes, it is important to ensure repeatability and uniform lubricant distribution, which also involves the degree of forging automation. Currently, in many die forges, lubrication is performed by a human operator, who often, despite their experience and good will, causes increased operation, and thus the lack of process repeatability and stability. The main disadvantage of this type of lubrication is non-uniform lubricant distribution causing a variable temperature distribution of the die and, as a result, accelerated local wear. Nevertheless, it is becoming more and more common to use partially or fully automated lubricating devices and systems, which enable repeatable and precise distribution of the lubricant to forging tools [13,14], which translates into reduced tool wear and the formation of defect-free forgings [15]. With the advancement of die forging process automation and robotization, more and more advanced lubricating and cooling systems are developed [16,17]. Moreover, thanks to the use of manipulators, fully flexible systems can be built allowing for controlling all important lubrication parameters, such as nozzle position, application time, the composition of the lubricant, etc. [18]. Furthermore, such solutions are fully synchronized with the operation of the forging unit, thus eliminating the human factor [19]. It should be noted that apart from the high costs of commercial solutions, the introduction of automated lubrication systems to hot die forging processes is difficult due to extreme operating conditions, including cyclic mechanical and thermal loads, as well as the short deformation time during forging of 0.2–0.5 s. It is much easier to use such solutions in pressing or semi-open cold die forging processes. Hence, there is much discussion in the extensive reference literature of lubrication systems in cold forging processes [20], or cold and warm forging [21,22], of mainly aluminum and its alloys [23], and definitely less for systems dedicated to warm and hot forging processes [24,25]. For example, in paper [1], one can find little information on lubrication systems and more on lubricant selection. In [26], one can find information on the devices and systems found in presses in the Japanese industry. The results of the European project described in [27] on the search for non-pollutants of the environment systems for the lubrication of tools in forging processes are also interesting. In [5], the research carried out by the team of scientists, based on experiments carried out during the manufacturing process, led to the development of guidelines, including a diagram for selecting optimal cooling–lubrication systems to increase the die service life. However, in [15], the factors affecting the lubrication quality and tool wear are described. In [28], the authors presented the results of modeling the quantity and direction of the lubricant portion. Unfortunately, despite repeated attempts to introduce automated lubrication systems to the forging industry, it involves quite high costs (several tens of thousands of euros for a lubrication system for one unit), which may not be set off when considering only the profit resulting from increased product quality and tool life. Therefore, an intensive search is currently taking place to find new technological solutions dedicated to specific forging applications to provide optimal tribological conditions with relatively low financial outlays, while being reliable and easy to implement in die forging shops. For example, in [29], the authors reviewed modern commercial lubrication systems and presented their proprietary low-cost lubricating and cooling device, the use of which resulted in the reduced forging tool wear. In [30], the authors developed a lubricating device equipped with a peristaltic pump, which ensured the delivery of a constant dose of lubricant in the front wheel forging process. However, [31,32] describe the use of an improved lubricating and cooling system additionally equipped with an automatic system cleaning system. The system was implemented in an industrial forging process of a forked type forging, which resulted in the tool life being extended by approximately 20% [33]. The analysis of the state of the art indicates that the issues of lubricating and cooling systems and process parameters of lubricant feeding are still valid. Therefore, conducting further research and development in this area is fully justified both from the scientific and financial point of view, because the issue of effective lubrication is still an unresolved problem and a considerable challenge for many research centers and industrial companies. The paper analyzes the results of the research on the effect of the dose (valve opening time and feeding pressure) of lubricant in laboratory conditions and then their verification in the industrial hot die forging process of high-nickel steel valve forgings in order to select optimal lubrication conditions and thus reduce die wear in use in the first forging operation.

## 2. Test Subject and Methodology

### 2.1. Research Problem Description

**Figure 1:**
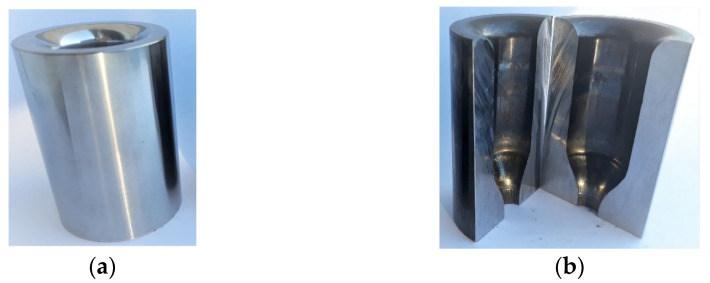
Selected dies used in the first operation (preliminary forging) of forging process of valve forgings: (**a**) new tool; (**b**) removed from production with visible wear.

The main research problem is the wear of the forging die (Figure 1), which is caused, among others, by improper lubrication in the forging process. For lubrication, a lubricant based on oil and graphite with the trade name FORG LUBE G25 (Industrial Solutions Group, Sroda Slaska, Poland) is used. The average life of the industrial forging tools analyzed is approximately 800 forgings. Long-term analyses of the process showed the lack of stability of the service life of these tools, which was probably caused by suboptimal tribological conditions. Long production cycles of up to approximately 2500 forgings, as well as short of 100 to 200 forgings, and complete die wear due to seizure after a few forgings were observed.

**Figure 2:**
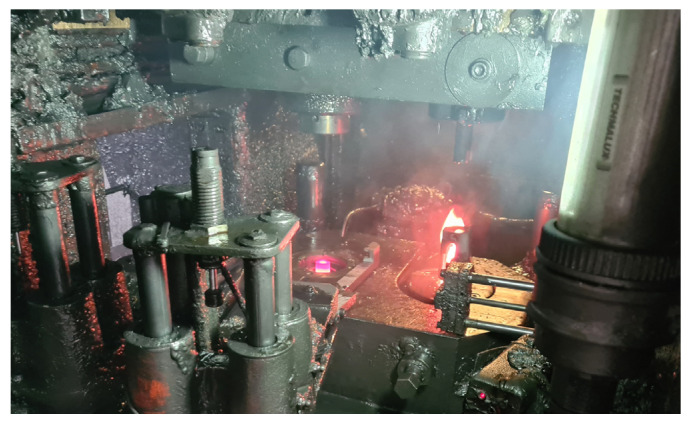
Photo of a press chamber in an industrial forging process with visible lubricant residues and a flame due to oil burnout after contact with a hot forging in the die.

Moreover, after the entire average production cycle, large amounts of lubricant residues were observed in the working cabin of the press, which would indicate incorrect settings of the lubricating and cooling system (Figure 2). Tests have shown that the best lubricating solution is an oil and graphite-based lubricant. Other solutions, e.g., based on a mixture of water and graphite, have not proved successful due to the different viscosities and boiling points of water and oil.

The whole forging process consists of two forging operations on a crank press with 7 MN [34,35]. A detailed analysis of the chosen die used in the first operation is shown in Figure 1. Such a die is used in the industrial manufacturing process of the valve forging of high-nickel high-temperature creep resisting steel (NCF 3015; BGH Company, Lugau, Germany). The analyzed type of forging is an important element of a motor engine, and due to the strict requirements for the automotive industry, it needs special supervision during the production process. The tools in the first operation are made of QRO 90 (Uddeholm, Mölndal, Sweden) steel thermally treated and gas nitrided (depth 0.2 mm, hardness 1200 HV). The chemical composition of this steel is presented in Table 1. After the finishing mechanical treatment, the quality of the impression surface, according to the manufacture technology, equals Ra = 0.32, and the hardness is at the level of 650 HV. In the industrial forging process, tools are heated to a working temperature of 250 °C. The charge material is in the form of a cylinder made of high-nickel steel heated to 1050 °C. The chemical composition of the input material and dies is given in Table 1.

Therefore, in order to comprehensively analyze the influence of the lubrication method on the wear of the forging die used to manufacture the valve forgings, the research was divided into two stages: die wettability tests in laboratory conditions (I) and verifying experimental tests in industrial hot die forging conditions (II).

### 2.2. The Lubricant Dosing Station Shako

**Figure 3:**
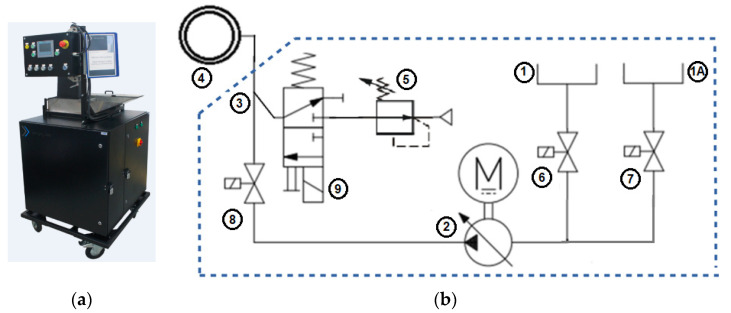
View: (**a**) the lubricating and cooling station, called “Shako”; (**b**) lubricant dosing system diagram, 1—lubricant tank, 1A—cleaning agent tank, 2—force pump, 3—tee, 4—head with nozzles, 5—pressure regulator, 6—cut-off valve of the liquid lubricant tank, 7—cut-off valve of the cleaning liquid tank, 8—cut-off, metering valve, 9—air valve of air exerting pressure on the liquid.

The tests were carried out at the lubricant dosing station named “Shako” (REBBMANN, Wroclaw, Poland) (Figure 3a), the simplified functional diagram of which is shown in Figure 3b. Until now, the industrial forging process used a fixed lubricating device with little adjustment capability, unlike the Shako system developed by the authors. The developed lubricating and cooling device, apart from the much greater adjustment capability compared to a fixed system, is equipped with a self-cleaning procedure. The existing lubricating device used in the industrial process operated at the maximum pressure of 4 bars, supplied from a system; however, as tests and observations have shown, it has not been verified whether this was the pressure actually applied to the lubricating head or a lower value due to lubricant graphite depositing in the lines, causing pressure losses and unstable tribological conditions. The clogging of lines and nozzles in the lubricating head has been repeatedly confirmed by press operators. For this reason, unlike previous solutions implemented by the authors, the Shako device was equipped with a special cleaning procedure, consisting of the periodic forced flushing of the system with oil from another tank, which significantly reduced the tendency for graphite deposition in the lines and clogging of the lubricating head nozzles, which affects the repeatability of the feeding of the set lubricant dose.

In addition, the Shako dosing station was used the first time for dosing lubricant in the form of oil with graphite. In previous applications, it was used to dispense water with graphite, which is characterized by a much lower viscosity. To meet this task, as a result of many tests, a pump was selected and used, which is able to pump the lubricant over a distance of 4 m.

The Shako dosing station measures the lubricant with a pump and has a cleaning procedure. The use of a dosing pump allowed eliminating the valve, the opening time of which determined the amount of lubricant supplied. A mechanical stirrer for the lubricating liquid has been added to the present device (in other applications, the so-called bubble gun was used) and the ability to set operating parameters—it does not have to rotate all the time, but it can be triggered for a given time from time to time.

The device also has a remote communication module, which greatly facilitates control, as it allows the possibility of remote change of parameters. Due to the cooperation of the developed device with other forging units, changes were also introduced in the control software.

### 2.3. Test in Laboratory Conditions 

**Figure 4:**
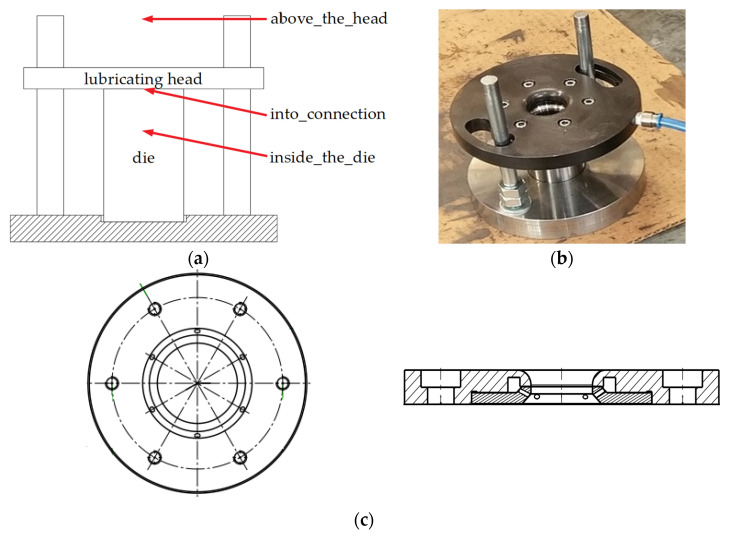
Test stand diagram: (**a**) schematic diagram; (**b**) test stand; (**c**) lubricating head diagram.

In these tests, die wettability tests in laboratory conditions (I), it was determined what lubricant amounts (depending on the settings of the lubricating device: the pushing pressure and the time of lubricant exposure were changed) remained inside the tool cavity during single lubrication, and which were falling out of the cavity due to incorrect settings of the lubricating device parameters. The tests were carried out on cold tools for different lubrication system settings (identical to the settings in the industrial process). The diagram of the test stand is shown in Figure 4. The test stand was built in such a way as to verify industrial conditions in which significant lubricant amounts, probably due to incorrect and variable settings, instead of falling directly and only into the die’s working cavity, bounced off or flew over the walls of the lubricating head. Such unstable conditions resulted in variable process tribological conditions, which in turn resulted in a high scatter of tool life in this operation, as well as their premature wear. The environmental aspect should also be noted, i.e., a considerable loss of lubricant, high contamination of the press chamber, self-ignition of lubricant oil causing an accumulation of smoke in the working area and, consequently, automatic stoppage of the manufacturing process.

Therefore, based on the analyses and preliminary tests in the industrial process, it was found that the lubricant, apart from its influx into the die cavity, often accumulated between the upper die surface of the lower surface of the lubricating head or completely escaped over the entire tool set, and based on this, the concept of the test stand was developed, which was to reflect the conditions of the industrial process (Figure 4). The amount of lubricant in each of the 3 zones was assessed by measuring the weight remaining on a tared paper towel sheet (Figure 4a): above the head; into connection; inside the die. For this purpose, a balance with an accuracy of 0.001 g was used. Due to the small amounts of lubricant supplied, each test (with the particular pressure and time parameters) was repeated 5 times, and based on this, the average values were determined.

After each test, each surface of the laboratory stand was thoroughly cleaned. In laboratory and industrial tests, the same lubricating head shown in Figure 4c was used. This head is characterized by six nozzles through which lubricant was pushed out. The nozzles in the form of holes are placed at an angle, thanks to which lubricant is supplied inside the die; they minimize the amount remaining on the connection of the lubricating head and the die.

To evaluate the results, it was assumed that the more lubricant remaining inside the tool cavity during laboratory tests, the better. It was so, because in the industrial process, rapid “seizing” of the working part of the tool cavity was observed, which was caused by an insufficient lubricant dose. Industrial process analysis results have shown that a significant portion of lubricant that should fall into the tool cavity is bounced off or sticks to the die walls or the lubricating nipple, rather than entering inside to improve lubrication. Furthermore, after the entire production cycle, huge amounts of lubricant residues were observed in the working space of the press, which would indicate that a large part of lubricant is sprayed out of the tools and wasted due to incorrect settings in the industrial process. Furthermore, due to the conditions prevailing in the industrial process, i.e., high temperature of the charge material and the working temperature of the tools, it was decided that after each test in laboratory conditions, complete cleaning of the system on the test stand would be tantamount to complete burnout of the remaining lubricant in the industrial process. This situation is confirmed by the appearance of a flame each time after lubricant is fed into the die in the industrial process (Figure 2).

### 2.4. Verification Tests in Industrial Conditions

The industrial conditions tests consisted in operating the dies used in the first forging operation of up to 500 forgings for various settings of the lubricating system parameters (similarly to the laboratory tests), the lubricant supply pressure, and the solenoid valve opening time, i.e., the lubricant exposure time, were changed. Then, for each setting variant, several forgings up to 500 pcs were cyclically collected from the process. This final explanation value was adopted, despite the fact that the average life was at the level of 900 pcs, for concerns that some of the variants may end prematurely and then it will not be possible to compare the results. Several forgings were obtained in this way for each variant. Measurements of each forging were made using 3D scanning, based on which changes in the geometry of the forgings were obtained in the form of a color deviation map. A graph with the wear curves for each variant of the lubrication settings was also developed illustrating the history of changes in the amount of material on the forgings, which, based on the inverse 3D scanning method developed by the authors, allowed assessing the degree of tool wear. Furthermore, for each variant, after 500 forgings were made, the dies were cut into two halves, and the working cavities were scanned, the working surfaces of the tools SEM were analyzed, and the geometric loss was determined in relation to the CAD model (Autodesk, San Rafael, CL, USA) of the unused tool. Based on this, the topography of each of the forgings was assessed as well as the progressive wear of the dies, visible, for example, in the form of longitudinal scratches or a slight increase in material on the forging, which indicates a loss of the tool’s material. Finally, to verify the results for the best variant of the lubricating and cooling system settings, the process of forging the valve forging for the maximum operation was carried out in order to assess the obtained results of both laboratory tests and short production runs and its suitability for normal production.

### 2.5. Measurement Methods Used

**Figure 5:**
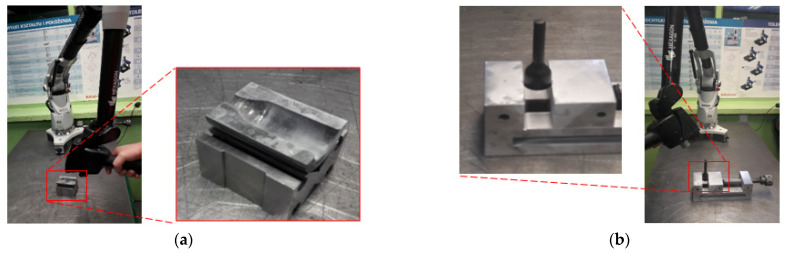
Station with measuring arm ROMER AbsoluteArm 7520si, integrated with a laser scanner RS3, used for the 3D measurements: (**a**) die measurement; (**b**) forging measurement.

For the tests, in order to measure the geometric changes of the working blanks of the analyzed dies and forgings (used in the 3D reverse scanning method), a measuring arm ROMER Absolute ARM 7520si integrated with a scanner RS3 (Hexagon Manufacturing Intelligence, Aarau, Switzerland) was used, as well as the Polyworks software (2015, InnovMetric Software Inc, Québec, Canada) and the Real Time Quality Meshing scanning technology. For the purposes of the tests, laboratory measurement stands were constructed, as shown in Figure 5. The device is assigned for measurements with the use of the laser scanner RS3 integrated with the arm, which provides the possibility of collecting up to 460,000 points/s for 4600 points on the line with the linear frequency of 100 Hz. The accuracy of the integrated scanning system SI according to the standard B89.4.22 equals 0.053 mm.

In order to conduct SEM analysis of the surface topography of the working cavities of the dies after different lubrication variants, we used the Tescan Vega 3 (Tescan Analytics, Brno, Czech Republic) scanning electron microscope equipped with detectors: SE, BSE, EDS, EDSD with a voltage of 30 kV and magnification up to 1 million times.

## 3. Discussion of Results

### 3.1. Laboratory Stage (I)

**Figure 6:**
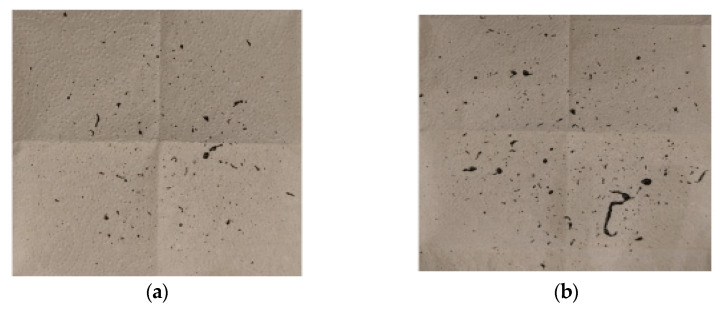

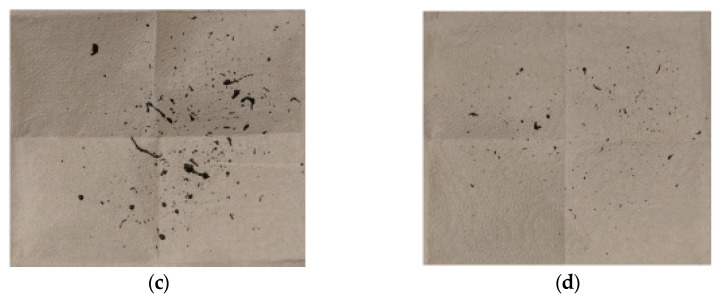
Sample photos of towels above the head for particular test parameters: (**a**) variant 1; (**b**) variant 3; (**c**) variant 2; (**d**) variant 4.

Laboratory tests included the following parameters of air pressure acting on the lubricant and the valve opening time presented in Table 2. Figure 6 shows examples of photos of paper towels above the head.

For variants 1 and 4, the amounts of lubricant bounced off the die were much smaller than for variants 2 and 3. The probable cause of the situation for variants 2 and 3 is the fact that not only is lubricant bounced off when entering the die, but also the agent that is dosed to the upper die part, which, combined with low dynamics (variant 2) and short time (variant 3), results in lubricant being bounced off the lubricant already fed into the die.

**Figure 7:**
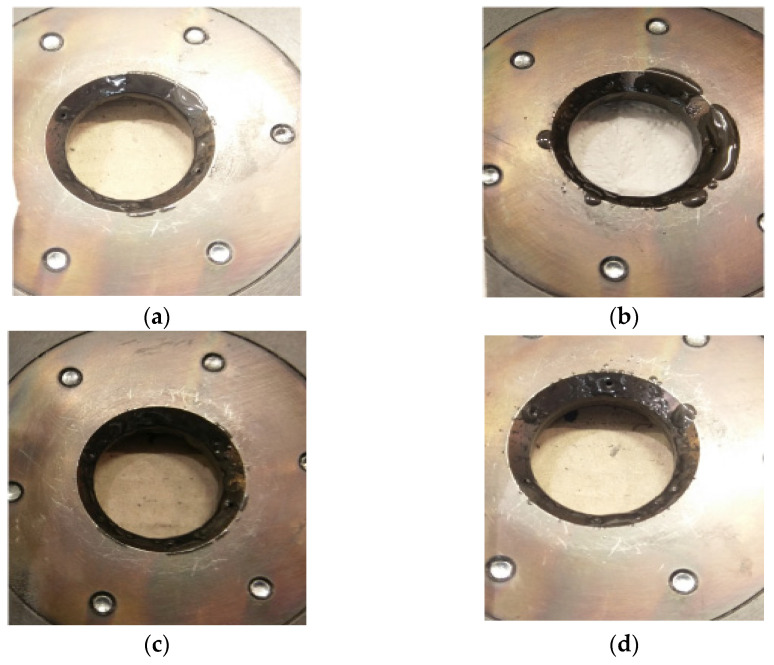
Lubricant remaining on the die for particular test parameters: (**a**) variant 1; (**b**) variant 3; (**c**) variant 2; (**d**) variant 4.

Figure 7 shows pictures of the lubricating nozzle with lubricant left over during the tests. Variants 1, 2, and 4 are very similar as opposed to variant 3. In variant 3, the accumulation of lubricant on the face of the nozzle head is clearly visible. 

This situation may be caused by liquid lubricant being pushed out from the nozzles in the initial phase in the form of a growing drop, which, after exiting the nozzle, “explodes” only to be subsequently delivered in the form of a continuous stream of liquid lubricant. Due to the short valve opening time (0.5 s in the 3 variant versus 0.7 s in the 4 variant), the lubricant deposited on the head as a result of the “explosion” is not taken toward the die due to the relationship between the viscosity and the further dose of lubricant, which takes place in variant 4. The situation of the “explosion” of drops is less noticeable in the 1 and 2 cases, where the dynamics of lubricant being pushed out is lower due to lower pressure.

**Figure 8:**
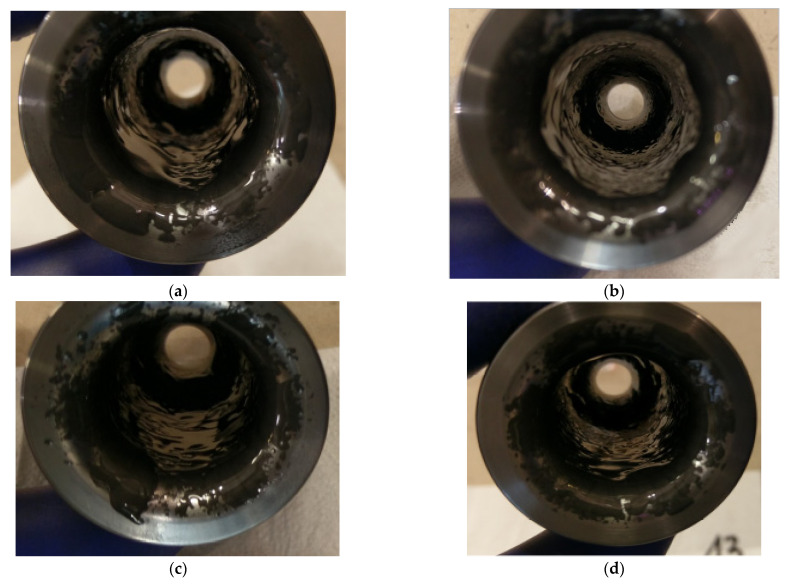
Lubricant fed inside the die for particular test parameters: (**a**) variant 1; (**b**) variant 3; (**c**) variant 2; (**d**) variant 4.

Figure 8 shows the inside of the die with the lubricant applied. The four variants were characterized by the lubricant fed the deepest into the constriction area of the die.

In the case of variants 1 and 2, the amounts of lubricant fed into the die were slightly smaller, but the vast majority of it was applied to the cylindrical part of the die above the constriction. In the case of variant 3, lubricant was mostly located on the cylindrical part, although much closer to the constriction as in the case of 1 and 2.

### 3.2. Tests in Industrial Conditions (II)

**Figure 9:**
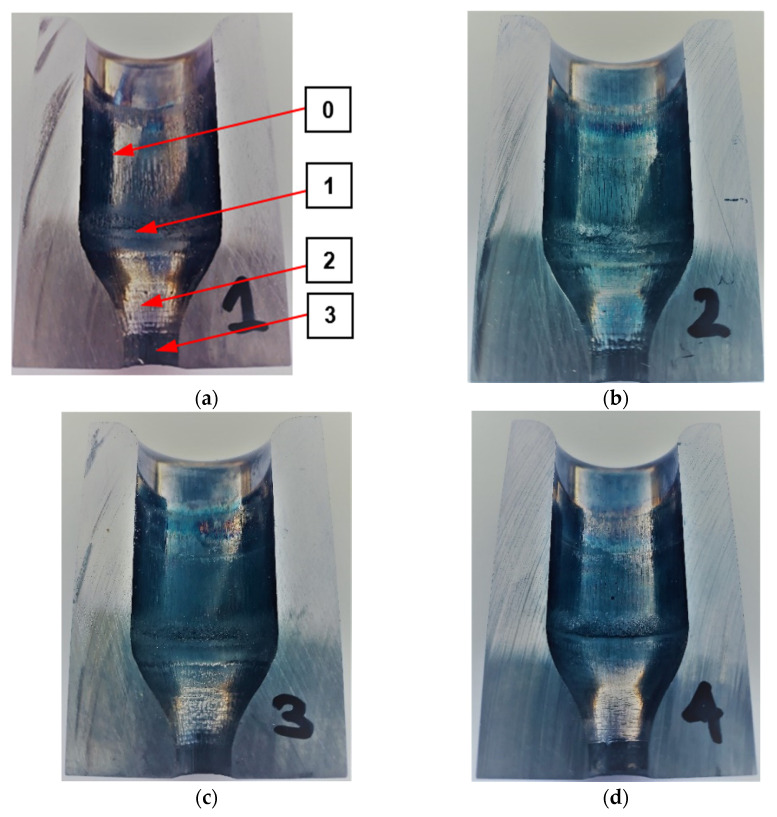
Macro photos of cut dies with marked all characteristic zones (in zone 0 the forging is not formed during 1st operation, zones 1-3 are involved in forming and wear, which affects the quality of the forging: (**a**) variant 1; (**b**) variant 2; (**c**) variant 3; (**d**) variant 4.

For the same settings of the lubricating and cooling system, verification tests were carried out in industrial conditions. Figure 9 shows macro photos of the dies cut in half after making 500 forgings for various settings of the lubrication system. A comparative analysis of the obtained results shows that all dies were worn in a similar way.

A detailed analysis of the obtained results is presented in Section 4.2.

## 4. Results

### 4.1. Analysis of Laboratory Stage Results (I)

Table 3 summarizes the results as mean values of five repeat tests for a particular variant (different valve opening and air pressure).

Based on the observation of lubricant distribution within the die and the numerical data, it was found that the most advantageous variant due to the amount of lubricant supplied into the die and wasted by being bounced off and deposited on the upper part of the die and the head is variant 4. In this variant, characterized by a pushing pressure of liquid lubricant at the level of 4 bar and a pushing time of 0.7 s, a relatively small amount of lubricant was found, which bounced off the die and was lost, adversely affecting occupational health and safety in industrial conditions (dirty work chamber, burning fumes) and having an undesirable environmental impact. In this case, slight lubricant deposition on the upper die surface and the lower surface of the lubricating head was also observed, which has a significant impact on the health, safety, environmental, and economic aspects described above. Variant 4 is also characterized by the most important parameter, namely the largest amount of lubricant supplied into the die of the cases under consideration. Moreover, it should be noted that in this variant, lubricant was fed the deepest into the die compared to the other variants. This is a very important feature, because the lubrication in the constricted die area has a key impact on the tribological conditions as well as tool wear and flaws occurring in the forging.

Then, to verify the obtained results, the lubrication system settings were implemented in the industrial process for short production runs of 500 forgings.

### 4.2. Results Analysis of Tests in Industrial Conditions (II)

**Figure 10:**
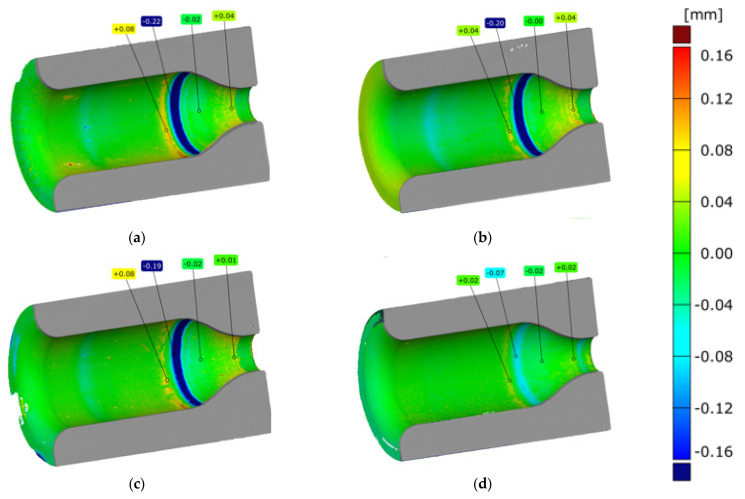
Results of 3D scanning of dies cut in half: (**a**) variant 1; (**b**) variant 2; (**c**) variant 3; (**d**) variant 4.

Analyzing the results concerning the wear of the tools shown in Figure 9, it can be seen that there are several wear zones in each of them. On the cylindrical side surface of the die cavity, a clear trace of the perimeter (edge after cutting) of the high-nickel steel preform, where the punch started upsetting the material, can be observed from the top (0). It should be emphasized that this zone of the die is an area that does not shape the forging and therefore does not affect its quality. This part of the forging after the first operation is formed during the second forging operation. Therefore, the possible wear of this zone (0) does not constitute a reason for withdrawing the die from further production. Slightly below, another circumferential mark can be seen on the cylindrical portion of the impressions (1), which is where the forging leg is pressed out after upsetting. Furthermore, there are longitudinal scratches in the direction of the punch movement and the flow of material for all the impressions. Some of the scratches are open, especially in the event of tools where lubricant is fed at a lower pressure (Figure 9a,b). For tools with higher lubricant supply pressure (Figure 9c,d), this area is smoother, although for all tools, slight longitudinal plastic deformations can be observed. Another characteristic zone is the transition area from the cylinder to cone (2); for all the tools, peripheral impressions are visible, which are the result of the lower bases of the incoming preforms being bounced off. In this area, there is a clear abrasive wear of the tool material, which can be seen even better in the tool scanning results. One can also see cracks slightly above this area, which are larger for tools with lower lubrication pressure and may be due to insufficient lubricant film in this area. The last characteristic zone is the area of cross-sectional area reduction, where the leg of the forging is created by concurrent extrusion (3). In this area, especially for tools with reduced lubrication pressure, characteristic thermomechanical crazing can be seen, which indicates the presence of high temperature during a long-time contact of the forging, extruded at high pressures, with the tool surface. The appearance of crazing may be indicative of a lack of lubricant in this area, which, in addition to lubrication, should insulate the cooler material of the tool from the hot forging. The least worn-out tool is the die, which worked with lubricant fed at a higher pressure and a longer exposure time, because it can be seen that in each of the areas, it is worn the least, and in the latter, it is difficult to notice traces of thermomechanical fatigue crazing, which indicates the best tribological conditions resulting from the most intensive lubrication. Figure 10 shows examples of scanning results of dies used in the first operation of preliminary roughing die forging of the valve forging after 500 forgings were made under different tribological conditions (four different lubrication system settings). Before scanning, the tools were cleaned of lubricant residues and scale. Then, they were scanned, and their digital images were converted into a cloud of points, turned into triangle meshes, and referenced to the nominal CAD model. The conducted analyses confirmed the tests carried out at an earlier stage (based on macro photos) and allowed for obtaining additional information on the size of geometry changes.

### 4.3. Results Analysis from the Measurement of Changes in the Geometry of Worn Dies Using 3D Scanning (II)

The comparative analysis of the results obtained (Figure 10) emphasizes that all the dies were worn in a similar manner, and several wear zones can be noticed in each of them. The perimeter preform trace (of the edge after cutting) of the high-nickel steel material (Figure 9) on the cylindrical side surface of the die cavity visible in earlier tests, where the punch started the upsetting of the material, is visible in the presented results (Figure 10) in the form of a light blue ring where the material loss is between −0.04 and −0.06 mm and in the case of the variant 4 cavity, the ring is not noticeable because the material loss is below −0.04 mm. In the next two areas, i.e., in the cylindrical impression, where, after upsetting, the leg of the forging is extruded, and in the area below the place where longitudinal scratches in the direction of the punch movement and material flow are visible in Figure 9, the material loss is below −0.04 mm, and it is visible for the scale adopted in Figure 10 in the form of a green area with a light blue tint. Another characteristic zone visible in Figure 9 is the area of the transition from cylinder to cone, which in Figure 10 is visible in the form of a ring indicative of wear at the level of −0.07 mm for variant 4 to even −0.22 mm for variant 1. The ring is formed in the place where the preform comes into contact with the tool for the first time, and its blue color is indicative of the amount of material loss. Furthermore, a second characteristic yellow ring is formed in front of this ring, which is indicative of cracks (visible in Figure 9) in this area. For tools with a lower lubrication pressure, the values are at the level of 0.08 mm, in the case of variant 3, they are at the level of 0.04, and they are the lowest for variant 4, where the value is at the level of 0.02 mm. The last characteristic zone is the area of reduction of the cross-sectional area, where the leg of the forging is created by concurrent extrusion and the material loss is very small, ranging from 0 to −0.02 mm. In the last part of the smallest diameter, grooves appear, which cause material growth of 0.02 to 0.04 mm.

**Figure 11:**
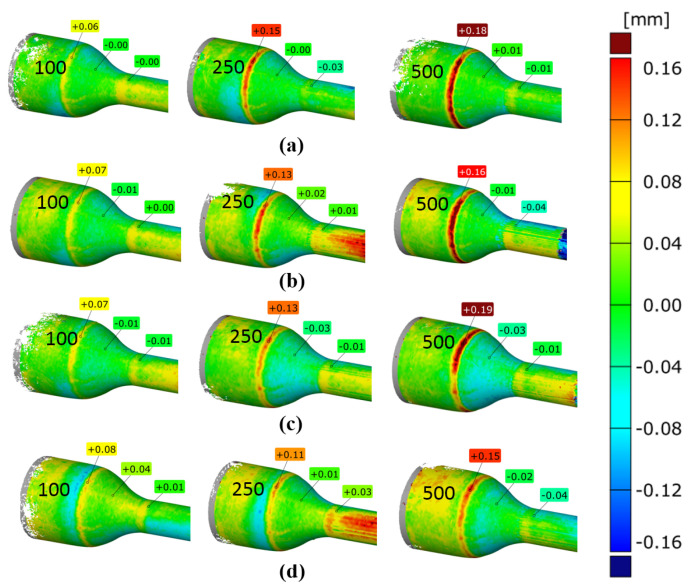
Three-dimensional (3D) scanning results of the next 100, 250, and 500 pieces of forgings: (**a**) variant 1; (**b**) variant 2; (**c**) variant 3; (**d**) variant 4.

Moreover, forgings were periodically sampled from the process to determine the intensity of geometry changes over time. Figure 11 presents selected color deviation maps of the 3D scanning results of periodically sampled forgings. These results make it possible to analyze the intensity of changes in the geometry occurring over time.

The analysis conducted shows that in the case of the variant 4 tool (Figure 11d) for the lubrication variant of 4 bars and 0.7 s opening time, these changes occur the slowest. This effect is visible in the form of the least visible red rings appearing on 250 forgings. For 500 forgings, this ring then develops unevenly around the circumference and obtains a value from 0.7 to 0.15 mm, which is indicative of material loss on the tool in the range of −0.07 to −0.15 mm. 

**Figure 12:**
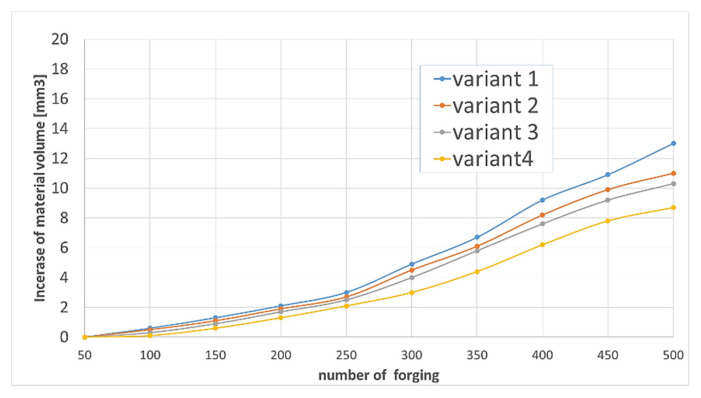
Scanning results of the cut dies for different pressure settings and solenoid valve openings.

In order to better visualize the volumetric changes in the subsequent forgings for each variant, the Polyworks software was used to build a graph of the volume changes of the forgings for the entire deformation area identical to the worn area of the die cavity (Figure 12), according to the 3D scanning revers method developed by authors [31,34].

Analyzing the gradual (as the number of forgings increased) increase in the volume of material on the forgings indicates the progressing wear (material loss) of each tool. Although these changes are small, as they only reach a few mm^3^ at the final stage of the service life, which is 500 forgings, an upward trend can be observed for each of the lubrication variants. The smallest increase in forging volumes, indicative of die wear, can be observed for tool representing variant 4.

### 4.4. The SEM Analysis of the Surface Topography of the Working Cavities of the Dies

**Figure 13:**
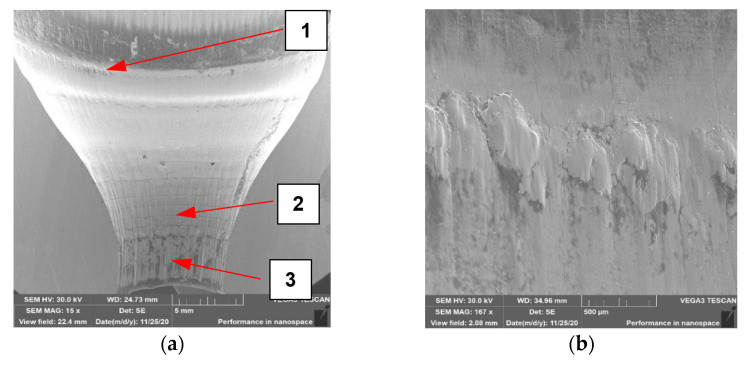

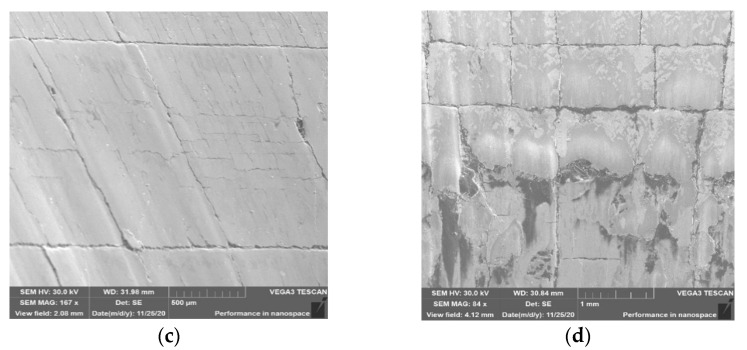
SEM analysis for tool 1: (**a**) photo of die cavity with marked characteristic zones formed forging in the 1st operation (in which appear different type of wear); (**b**) area in zone 1; (**c**) area in zone 2; (**d**) area in zone 3.

To carry out the SEM analysis, due to the fact that the main part forming the forging, which is also the most worn out, each of the dies was cut from the bottom (the smallest diameter being the area forming the forging leg) up to a height of 30 mm. Figure 13 shows the results of SEM surface topography analysis for each of the four dies. Additionally, photos b to d (beside and below) show the enlarged characteristic tool areas. The analysis of the surface topography for the tool No. 1 (Figure 13) shows that in area 1 of the lower base of the preform, bouncing off causes intensive abrasion of the tool material (Figure 13a). In this area, the scanning results showed material loss in the normal direction, and just above it (dark area) showed material growth, which may be the result of lubricant graphite sticking and adhering to the tool surface.

**Figure 14:**
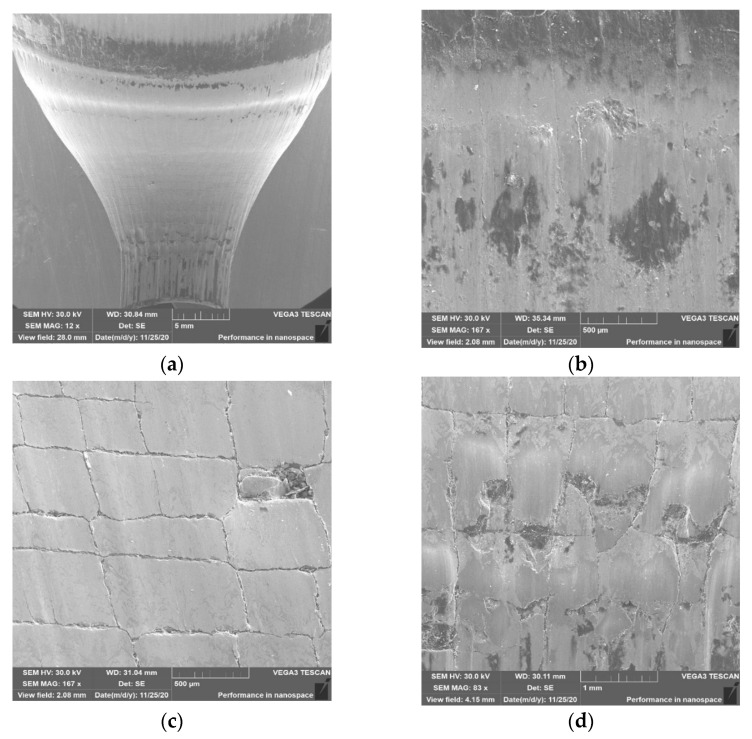
SEM analysis for tool 2: (**a**) cavity; (**b**) area in zone 1; (**c**) area in zone 2; (**d**) area in zone 3.

The loss of material in zone 1 may be the result of repeated impact of the preform with sharp edges in the lower base and tearing out (Figure 13b) along with plastic deformation of small fragments of the tool material. A more thorough organoleptic analysis showed a clear step in this area, which is indicative of the occurrence of plastic deformation. In zone 2, the primary thermomechanical crazing and the beginnings of the formation of the secondary crazing are visible (Figure 13c). In zone 3, thermomechanical crazing that is rubbed and torn in the direction of the material flow can also be observed. There is clearly a lack of sufficient amount of lubricant in zones 2 and 3, as evidenced by the formation of crazing and material abrasion. The detachable small hard particles of the material from zone 2 may cause the formation of longitudinal scratches on forgings, which can be seen particularly well in Figure 13d and has been confirmed by tests using 3D scanning. The topography analysis for die no. 2 (Figure 14) showed the presence of similar phenomena in the selected three zones.

**Figure 15:**
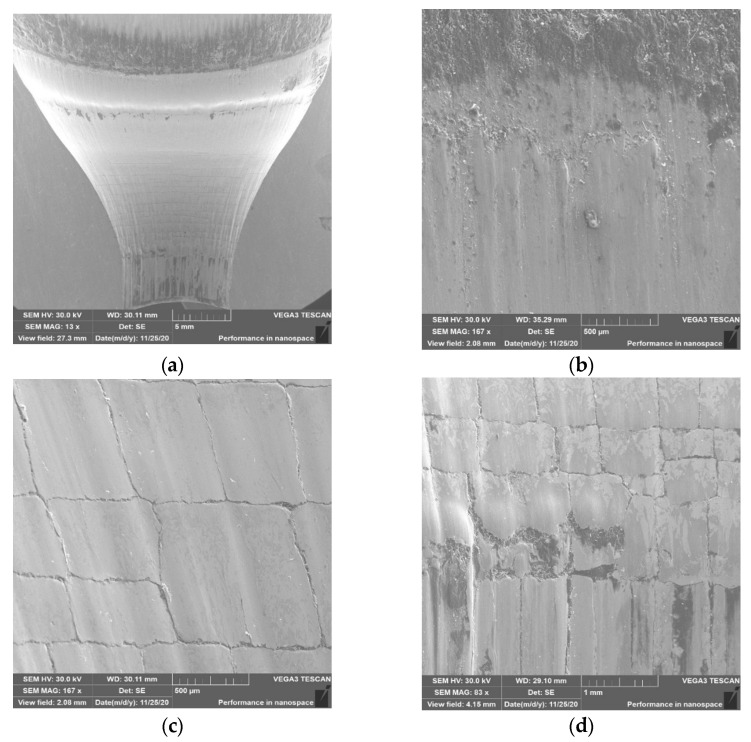
SEM analysis for tool 3: (**a**) cavity; (**b**) area in zone 1; (**c**) area in zone 2; (**d**) area in zone 3.

The thermomechanical crazing in zones 2 and 3 (Figure 14c) is slightly less visible, which may be indicative of slightly better tribological conditions in this case. In zone 3, we can see alternate tearing out of “square” parts of the crazing as material sticking progresses (Figure 14d). Lubricant remnants can also be observed in some of the torn areas. The analysis of the SEM results for the 3rd tool (Figure 15a) is almost identical to that observed for the 2nd die. It can be seen that the topography appears to be slightly softer for tool 2, which may be due to narrowly better lubrication conditions. This is also evidenced by the slightly larger thermomechanical crazing in zone 2 (Figure 15c) and a narrowly smaller number of tears, places of material sticking, and lubricant residues.

The greater amount of lubricant in this case may result in better thermal protection of the tool against the hot forging and a certain shift in time of the destructive mechanisms causing thermal and thermomechanical fatigue on the surface of the tool cavity.

**Figure 16:**
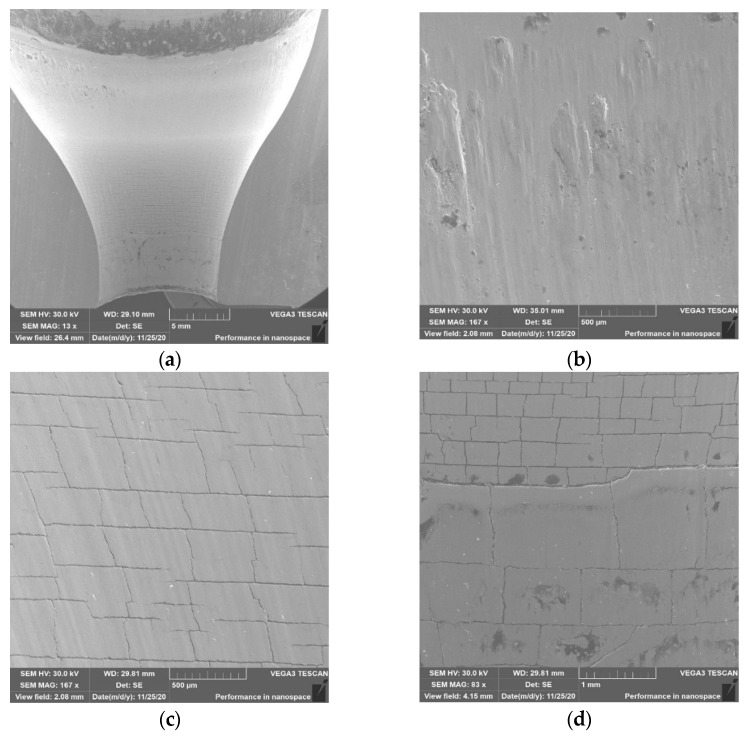
SEM analysis for tool 4: (**a**) cavity; (**b**) area in zone 1; (**c**) area in zone 2; (**d**) area in zone 3.

In the case of the last tool no. 4 (Figure 16), it can be observed that it is the least worn in all analyzed zones compared to the other three dies. In area 1, there is no visible change in the geometry due to plastic deformation, which may be the result of the most advantageous lubrication and on the one hand, better flow of the forging material, and on the other hand, protection of the lubricant against hitting the base of the preform and from contact and overheating of this area with the hot charge material. 

Areas 2 and 3 show characteristic thermomechanical crazing, but it is free of chipping and sticking, especially in area 2 (Figure 16c). It can also be seen that the crazing is not yet fully formed, as some of the lines are not fully connected. However, in the case of area 3, it can be observed that at the top, the thermomechanical crazing is smaller than below. It is difficult to explain this situation and find its cause. Nevertheless, when analyzing the wear for tool 4, it can be concluded that the most favorable tribological conditions prevailed in the forging process, which were caused by an increased presence of a lubricant. Moreover, the higher pressure and longer lubricant supply time made it reach the lower parts of the die cavity and provided better lubrication, which translated into reduced wear and the formation of forgings without longitudinal scratches on the stem.

### 4.5. The Results Analysis of Forgings Change Geometry with 3D Reverse Scanning Method (II)

**Figure 17:**
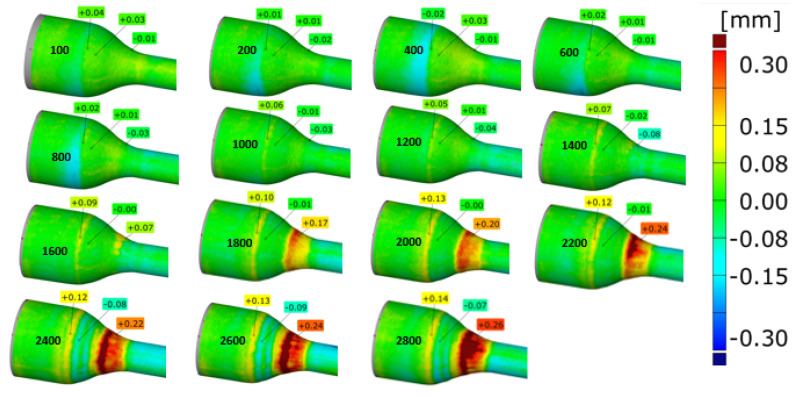
Comparison of the scans of forgings in the form of a change in the shape of the selected area in respect of the 100th forging, from 500 to 2800 items.

To confirm the completed laboratory tests, and subsequent tests in the industrial conditions of short production runs, a decision was made to perform industrial forging of a much longer run until the tool’s complete deterioration. The settings of the lubricating and cooling system were adopted as for tool 4, i.e., 4 bars and 0.7 s of lubricant exposure time. The production process standard settings are most often 4 bars with the exposure time of 0.5s. Figure 17 show the results of 3D scanning of forgings (every 100th item) in the form of a color map of deviations describing the changes in the shape of the selected surface in respect of the scan of the 100th forging, which were obtained according to the measurement technique presented above.

**Figure 18:**
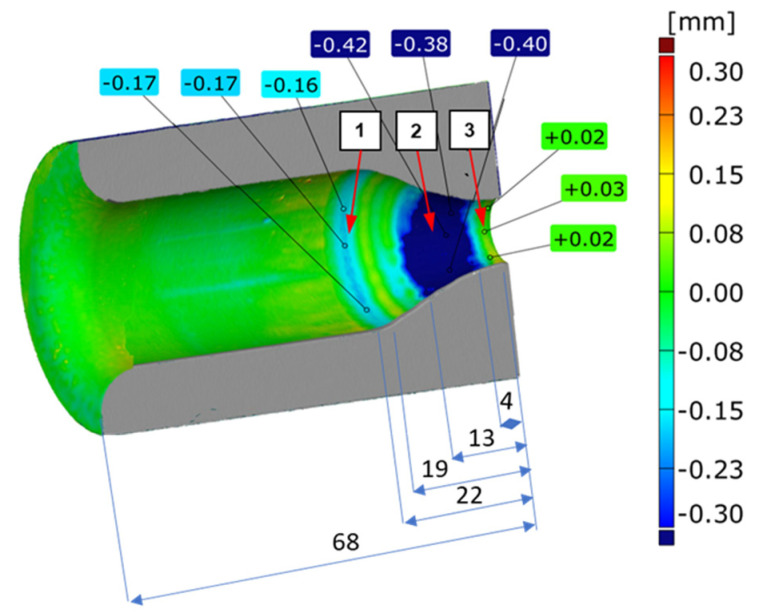
View of a scan results of the die after 2830 forgings with the characteristic zones (detailed analyzed and showed in Figure 13a with the lengths of: the die height and the individual zones are given in mm.

The results suggest that with the increasing number of forgings, a progressing wear of the tool is observed. According to the idea of the 3D method, a geometric growth of the material on the forging can be seen, which is mostly localized in the area of the stem (the most deformed part of the forging) in the form of long longitudinal groove-like scratches (Figure 17). In turn, slightly above this zone (on the profile), we can observe a growth of material on the forging together with the increasing number of forgings, pointing to a loss of material on the tool, and it is possible to determine the history of the wear performance of the die as well. A more complex macro-analysis as well as the use of FEM (high values of normal pressures and a long contact time were observed) made it possible to establish that initially, this area was dominated by plastic deformation, which in time transformed into thermomechanical fatigue and abrasive wear. Based on the above results, Figure 18 shows the results of 3D scanning of the analyzed die after 2830 forgings together with the marked zones, in which we can distinguish between the dominant destruction mechanisms, which will be identified and confirmed by further research.

**Figure 19:**
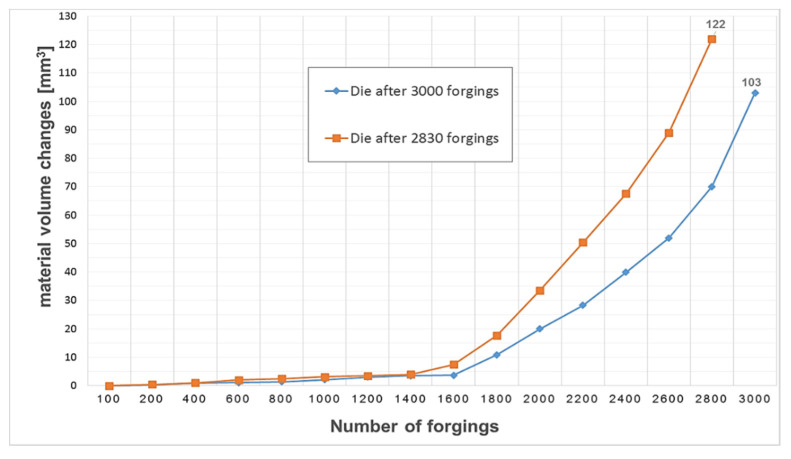
Volume changes–loss of material for the selected tools calculated from forgings.

Additionally, in order to determine the changes and their intensity during the entire operation of the selected tools, an analysis of the volumetric changes of the material was carried out, using cyclically collected forgings from the process. It should be noted that the tests for the same settings were repeated once again, and even a slightly higher operation of 3000 forgings was achieved (Figure 19).

When analyzing the graph, it can be noticed that the exploitation process is characterized by an increase in the material on the forging, which, based on the reverse scanning method developed by the authors, means that in dies cavities, there is a loss/wear of material. This phenomenon is more intensified, and the material loss process increases significantly after the production of 1600 forgings, until a loss of 122 mm^3^ is obtained for die after 2830 forgings and up to about 103 mm^3^ for tool after 3000 pieces.

## 5. Conclusions

The study discusses the analysis of the influence of the lubricant dose (through changes in pressure and solenoid valve opening time) on the lubrication conditions and the forging die wear. Observation and analysis of the industrial forging process of the valve forging showed that due to improper settings of the dosing parameters in the current lubrication system, a significant part of graphite lubricant fails to reach the tool cavity. Moreover, the analysis showed that the actual lubricant doses supplied have different volumes due to the lack of control of the lubricating system parameters and the system cleaning procedures. Unstable and uncontrolled lubrication conditions cause variable tribological conditions, which results in uneven die wear and high total lubricant consumption. The authors used the lubricating and cooling system developed by them, having automatic cleaning procedures of the entire system, as opposed to a fixed lubrication system with standard adjustment capabilities, which is used in the industrial process. In the tests divided into two stages, tests were first carried out in laboratory conditions to determine the highest wettability and lubricant dosing into the tool cavity. Subsequently, to verify the obtained results, tests were carried out under industrial forging conditions for short production runs of up to 500 forgings. The most advantageous variant of the pressure setting of 4 bars and the solenoid valve opening time of 0.7 s, confirmed based on short production runs, was implemented in the normal production process, which allowed for the maximum tool use with over 2830/3000 correct forgings made. The obtained results indicate that by properly selecting the parameters, which may have to be determined individually for each process, e.g., the pressure value and the lubricant exposure time, as well as the implementation of cleaning procedures, forging tool wear can be reduced. The environmental aspect touched upon in the paper is also worth noting, because changes in the settings of the device influenced the efficiency, as the vast majority of lubricant fell into the die cavity, which reduced the previous lubricant misuse and contamination of the press chamber. The approach proposed in the paper is one of many, because, as the state of the art shows, many factors affect the lubrication quality, indicating the use of another, more optimal lubricant, or changing the lubrication technology and design of lubricating devices. Being aware of this fact as well as having experience in this matter, further research directions to be taken are changing the structure of the lubricating ring and developing another (hydraulic-pneumatic) lubricant dosing system, taking into account the lubrication sequence divided into cycles (blowing off the scale with air, spraying lubricant mist, drying, etc.).

## Figures and Tables

**Table 1:** The chemical composition of input material (NCF3015) and material of forging dies (QRO 90) (%).

Material/Elements	C	Si	Mn	P	Ti	Ni	Mo	Al	Cr	Nb	Cu	V
**NCF 3015**	0–0.08	0–0.50	0–0.50	0–0.015	2.30-2.90	30–33.50	0.4–1.00	1.6–2.20	13.5–15.5	0.40–0.90	max. 0.5	-
**QRO 90**	0.38	0.30	0.75	-	-	-	2,25	-	2.6	-	-	0.9

**Table 2:** Designation of tests depending on the parameters.

Valve Opening Time	Air Pressure	
	3 (bars)	4 (bars)
0.5 s	(1)	(3)
0.7 s	(2)	(4)

**Table 3:** Average of five tests of the lubricant amount (in grams) deposited in selected areas for the variants of pressure and the time of its exertion under consideration.

Zone of Measurements	Valve Opening Time (s)	Air Pressure	
		3 (bar)	4 (bar)
**Above the Head**	0.5	0.014	0.029
	0.7	0.028	0.013
**On the Connection**	0.5	0.120	0.146
	0.7	0.110	0.116
**Inside the Die**	0.5	0.555	0.513
	0.7	0.551	0.560

## Data Availability

Data sharing not applicable.
